# In Vitro Antiviral Activity of a New Indol-3-carboxylic Acid Derivative Against SARS-CoV-2

**DOI:** 10.32607/actanaturae.26623

**Published:** 2023

**Authors:** A. N. Narovlyansky, M. V. Filimonova, N. G. Tsyshkova, A. V. Pronin, T. V. Grebennikova, E. V. Karamov, V. F. Larichev, G. V. Kornilayeva, I. T. Fedyakina, I. V. Dolzhikova, M. V. Mezentseva, E. I. Isaeva, V. V. Poloskov, L. S. Koval, V. P. Marinchenko, V. I. Surinova, A. S. Filimonov, A. A. Shitova, O. V. Soldatova, A. V. Sanin, I. K. Zubashev, A. V. Ponomarev, V. V. Veselovsky, V. V. Kozlov, A. V. Stepanov, A. V. Khomich, V. S. Kozlov, S. A. Ivanov, P. V. Shegai, A. D. Kaprin, F. I. Ershov, A. L. Gintsburg

**Affiliations:** National Research Centre for Epidemiology and Microbiology named after the honorary academician N.F. Gamaleya, Ministry of Health of the Russian Federation, Moscow, 123098 Russian Federation; National Medical Research Center for Radiology, Ministry of Health of the Russian Federation, Obninsk, 249036 Russian Federation; N.D. Zelinsky Institute of Organic Chemistry, Russian Academy of Sciences, Moscow, 119991 Russian Federation; Gamasintez LLC, Moscow, 123098 Russian Federation

**Keywords:** SARS-CoV-2, indole-3 carboxylic acid derivative, antiviral activity, cell culture

## Abstract

The coronavirus disease (COVID-19) pandemic has brought into sharp relief the threat posed by coronaviruses and laid the foundation for a fundamental analysis of this viral family, as well as a search for effective anti-COVID drugs. Work is underway to update existent vaccines against COVID-19, and screening for low-molecular-weight anti-COVID drug candidates for outpatient medicine continues. The opportunities and ways to accelerate the development of antiviral drugs against other pathogens are being discussed in the context of preparing for the next pandemic. In 2012–2015, Tsyshkova et al. synthesized a group of water-soluble low-molecular-weight compounds exhibiting an antiviral activity, whose chemical structure was similar to that of arbidol. Among those, there were a number of water-soluble compounds based on 5-methoxyindole-3-carboxylic acid aminoalkyl esters. Only one member of this rather extensive group of compounds, dihydrochloride of 6-bromo-5-methoxy-1-methyl-2-(1-piperidinomethyl)-3-(2-diethylaminoethoxy) carbonylindole, exhibited a reliable antiviral effect against SARS-CoV-2 *in vitro*. At a concentration of 52.0 μM, this compound completely inhibited the replication of the SARS-CoV-2 virus with an infectious activity of 106 TCID50/mL. The concentration curves of the analyzed compound indicate the specificity of its action. Interferon-inducing activity, as well as suppression of syncytium formation induced by the spike protein (S-glycoprotein) of SARS-CoV-2 by 89%, were also revealed. In view of its synthetic accessibility − high activity (IC_50_ = 1.06 μg/mL) and high selectivity index (SI = 78.6) − this compound appears to meets the requirements for the development of antiviral drugs for COVID-19 prevention and treatment.

## INTRODUCTION

(WHO) declared “an end to COVID-19 as a public health emergency” [1]. Just like that, the pandemic that lasted 3 years 1 month and 24 days was over. According to the WHO, globally, as of July 12, 2023, there had been 767,972,961 confirmed cases of COVID-19, including 6,950,655 deaths. A total of 22,967,718 confirmed cases of COVID-19 and 399,715 deaths have been documented in Russia [2]. However, even according to WHO estimates, the number of COVID-19 deaths exceeds 20 million people [3].

Although the end of the pandemic and decline in the total number of infection cases have been proclaimed, the COVID-19 epidemic cannot be considered to have completely subsided. New subvariants of the virus (XBB.1.16 and XBB.2.3) have emerged; that is why research that aims to update existing COVID-19 vaccines and search for small-molecule anti-COVID-19 drug candidates for outpatient use continues to this day. Furthermore, approximately 65 million patients have been identified as suffering from long-term sequelae of the SARS-CoV-2 infection. These cases are referred to as “post COVID-19 conditions” or “long COVID” [4].

The pandemic of the COVID-19 coronavirus disease has given us a new appreciation of the threat posed by coronaviruses and has spurred a fundamental analysis of this viral family, as well as a search for effective anti-COVID drugs. Obviously, efficient therapeutic strategies for COVID-19 are still needed. A number of antiviral drugs such as remdesivir, nucleoside inhibitors (AT-527 and molnupiravir), the main protease (Mpro) inhibitor nirmatrelvir, the nirmatrelvir–ritonavir combination and molnupiravir, and immunotropic drugs (baricitinib, tocilizumab, and corticosteroids, etc.) were tested during the pandemic phase [5]. However, almost no effective small-molecule oral antivirals have been developed for outpatient therapy [6].

In 2012–2015, Tsyshkova et al. synthesized a group of water-soluble low-molecular-weight compounds exhibiting an antiviral activity, whose chemical structure was similar to that of arbidol. Among those, there are a number of compounds based on 5-methoxyindole- 3-carboxylic acid aminoalkyl esters [7]. Only one member of this rather extensive group of compounds exhibited a reliable antiviral effect against SARS-CoV-2 *in vitro*; this compound was investigated in this study.

## EXPERIMENTAL

**Fig. 1:**
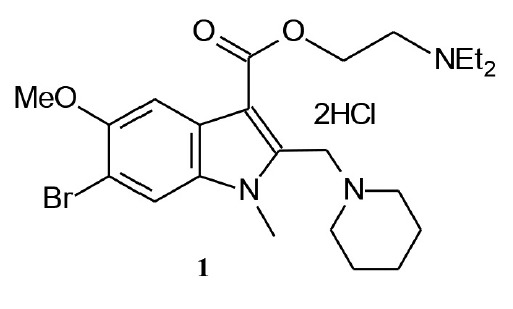
6-Bromo-1-methyl-5-methoxy-2-(1-piperidinomethyl)- 3-(2-diethylamino ethoxy)carbonylindole dihydrochloride (compound **1**)

**The study compound**, dihydrochloride of 6-bromo- 5-methoxy-1-methyl-2-(1-piperidinomethyl)-3-(2-diethylaminoethoxy)carbonylindole (**1**) (*Fig. 1*), was synthesized [7] at the National Medical Research Center for Radiology of the Ministry of Health of the Russian Federation.

**Fig. 2:**
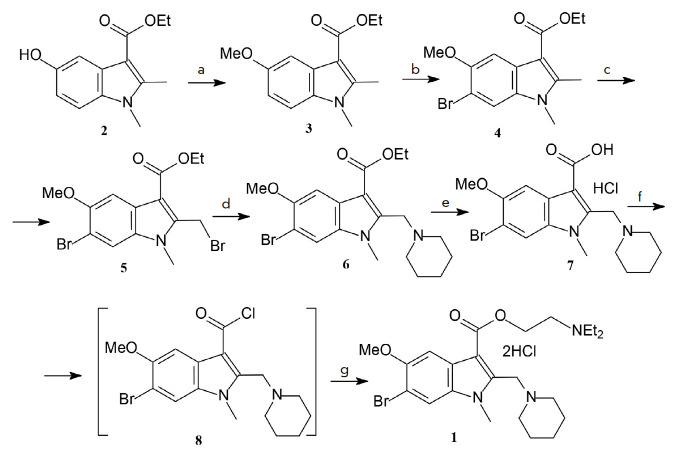
Reagents and conditions: a. (1) aq. NaOH, dioxane; (2) Me2SO4, 20°C; b. N-bromosuccinimide, CCl4, boiling; c. N-bromosuccinimide, (PhCOO)2, CCl4, irradiation (100 W bulb), boiling; d. piperidine, PhH, 20°C; e. (1) aq. NaOH, EtOH, boiling; (2) HCl (conc.); f. SOCl2, dioxane, DMFA (cat.), 60°C; g. (1) Et2NCH_2_CH_2_OH, Et3N, PhH, boil.; (2) HCl, Et2O, acetone, 20°C

Compound **1 **was obtained via multistep synthesis (the scheme is shown in *Fig. 2*).

The solvents and reagents used in this study, including ethyl-5-hydroxy-1,2-dimethyl-1*H*-indole-3- carboxylate, were purchased from Acros Organics. The melting points were measured using a Kofler heating bench. The IR spectra were recorded on a Bruker ALPHA T FT-IR spectrometer. The 1H NMR spectrum of the solution in DMSO-*d*6 was recorded on a Bruker AC-200 spectrometer at 298 K. The mass spectrum (EI) was recorded on a SHIMADZU LCMS- 8040 mass spectrometer using a direct-insertion probe in the positive ion scanning mode (Q3+Scan).

**1,2-Dimethyl-5-methoxy- 3-(ethoxycarbonyl)indole (3)**

A NaOH solution (10%, 40.0 mL) was added to a solution of 4.66 g (0.02 mol) of compound **2 **in 40.0 mL of dioxane at 20°C; then 4.0 mL of dimethyl sulfate (0.042 mol) was added dropwise. The reaction mixture was stirred for 2 h, diluted with water, and cooled (to 4°C). The precipitate was filtered off, washed with water, and vacuum-dried (2 torr) over P2O5. Compound **3 **(4.65 g, 94%) was obtained in the form of crystals with *T*m = 113°C (in the literature,* T*m = 117.5–118°C [8]).

**6-Bromo-1,2-dimethyl-5-methoxy- 3-(ethoxycarbonyl)indole (4)**

A mixture of 4.65 g (0.0188 mol) of compound **3 **and 3.36 g (0.0188 mol) of N-bromosuccinimide in 75.0 mL of CCl4 was heated for 5 h upon boiling. The precipitate (succinimide) was filtered off from the hot reaction mixture. The filtrate was concentrated (by 1/3) by boiling away the solvent and cooling. The precipitate was filtered off, washed with CCl4 on a filter, and vacuum- dried (2 torr). Compound **4 **(3.3 g, 54%) was obtained in the form of crystals with *T*m = 156°C (in the literature, *T*m = 164–165°C [8]).

**6-Bromo-2-bromomethyl-5-methoxy- 1-methyl-3-(ethoxycarbonyl)indole (5)**

A mixture of 3.3 g (0.0101 mol) of compound **4**, 1.81 g (0.0101 mol) of N-bromosuccinimide, and 0.1 g of benzoyl peroxide in 30.0 mL of CCl4 was boiled under illumination with a 100 W bulb during 5 h. After the succinimide solution had been filtered off from the hot mixture and the filtrate had cooled (to 20°C), the precipitate was filtered off, washed with CCl4 on a filter, and vacuum-dried (2 torr). A total of 3.16 g (78%) of compound **5 **was obtained in the form of crystals with* T*m = 142°C (in the literature, *T*m = 141–142°C [8]).

**6-Bromo-5-methoxy-1-methyl- 2-( 1-piperidinomethyl)-3-(ethoxycarbonyl)indole (6)**

A solution of 4.0 g (0.01 mol) of compound **5 **and 1.7 g (0.02 mol) of piperidine in 50.0 mL of benzene was left to rest at room temperature for 12 h. The precipitate (piperidine bromohydrate) was filtered off; the filtrate was concentrated to dryness under vacuum. Crystallization of the precipitate from ethanol yielded 1.7 g (82.9%) of compound **6 **as crystals with* T*m = 124–125°C (in the literature, *T*m = 124–125°C [8]). Anal. Calcd. for C19H25BrN_2_O3: C, 55.75; H, 6.16; N, 6.84. Found: C, 55.72; H, 6.20; N, 7.02.

**Hydrochloride of 6-Bromo-5-methoxy-1-methyl- 2-(1-piperidinomethyl)-indole-3-carboxylic acid (7)**

A solution of 6.0 g (0.15 mol) NaOH and 4.1 g (0.01 mol) of compound **6 **in 60.0 mL of ethanol and 3.0 mL of water was boiled for 3 h. The reaction mixture was cooled down, diluted with water (10 mL), and acidified with concentrated hydrochloric acid. The precipitate was filtered off, washed with water on a filter, and vacuum-dried (2 torr) over P2O5. A total of 4.10 g (98%) of compound **7 **in the form of crystals with *T*m = 236–238°C was obtained. Anal. Calcd. for C17H22BrClN_2_O3: C, 48.88; H, 5.31; N, 6.71. Found: C, 48.68; H, 5.32; N, 6.65.

**Dihydrochloride of 6-bromo-5-methoxy- 1-methyl-2-(1-piperidinomethyl)- 3-(2-diethylaminoethoxy)carbonylindole (1)**

Thionyl chloride (3.0 mL, 0.041 mol) and 2 droplets of dimethylformamide were added to a solution of 1.67 g (0.004 mol) of hydrochloride **7 **in 30.0 mL of dioxane under stirring. The reaction mixture was heated to 60°C during 3 h, concentrated to dryness under vacuum, and the remaining mixture was washed with diethyl ether. The resulting powdered chloroanhydride **8** was dissolved in 25 mL of benzene without additional purification and treated with a mixture of 1.2 mL (0.008 mol) of N-diethylaminoethanol and 1.12 mL (0.008 mol) of triethylamine. The reaction mixture was heated to 80°C during 2 h and cooled down. The precipitate (triethylamine hydrochloride) was filtered off and washed with hot benzene. The pooled filtrate was concentrated to dryness under vacuum; the remaining mixture was washed with hexane and vacuum- dried. A diethyl ether solution of hydrogen chloride (~ 30%, 2 mL) was added to the solution of the resulting product in 10 mL of acetone. The reaction mixture was concentrated to dryness under vacuum; the remaining mixture was crystallized from 2-propanol. Compound **1 **(1.9 g, 85.2%) with *T*m = 237–240°C was obtained. Its physicochemical characteristics are described below.

**IR (KBr, ν, cm-1)**

**Fig. 3:**
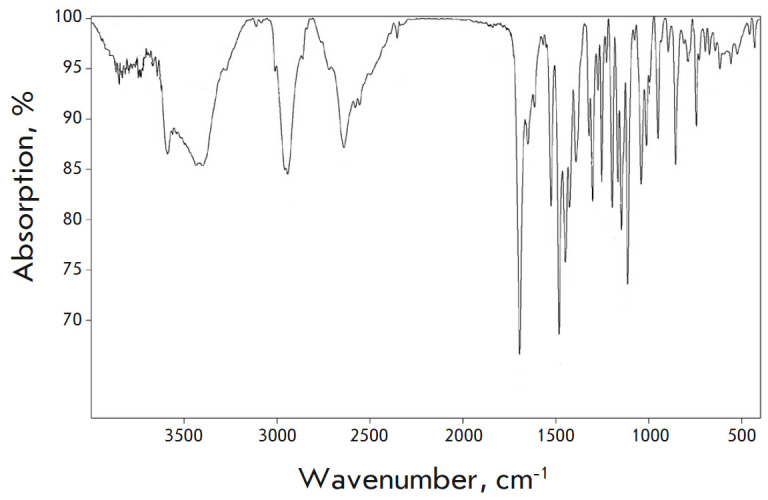
IR spectrum of compound **1**

859, 1041, 1114, 1148, 1197, 1303, 1393, 1426, 1449, 1483, 1650, 1694 (C=O), 2354–2700, 2942, 3397, 3588 (*Fig. 3*).

**1H NMR spectrum (200 MHz, DMSO)**

**Fig. 4:**
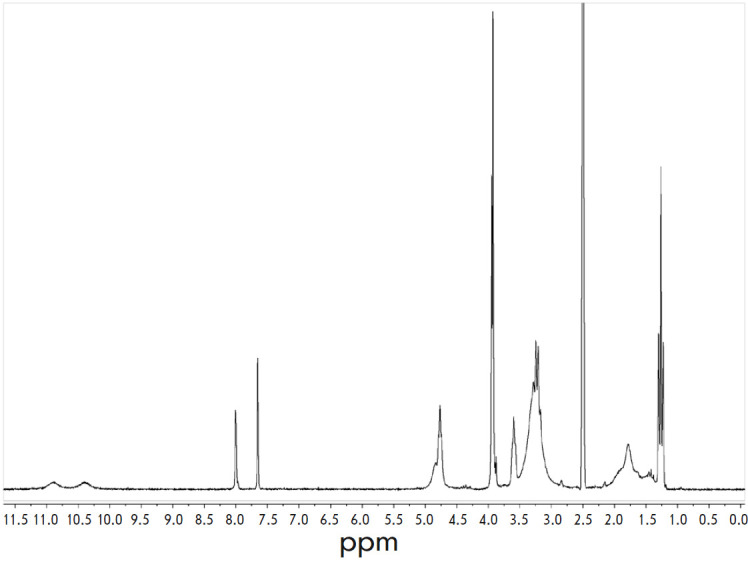
^1^H NMR spectrum of compound **1**

δ 10.76 (br s, 1H), 10.23 (br s, 1H), 8.03 (s, 1H), 7.65 (s, 1H), 4.87 (d, *J *= 4.8 Hz, 2H), 4.76 (t, *J *= 5.1 Hz, 2H), 3.95 (s, 3H), 3.93 (s, 3H), 3.61 (m, 2H), 3.50–3.06 (m, 8H), 2.15–1.33 (m, 6H), 1.26 (t, *J *= 7.2 Hz, 6H) (*Fig. 4*).

**Mass spectrum**

**Fig. 5:**
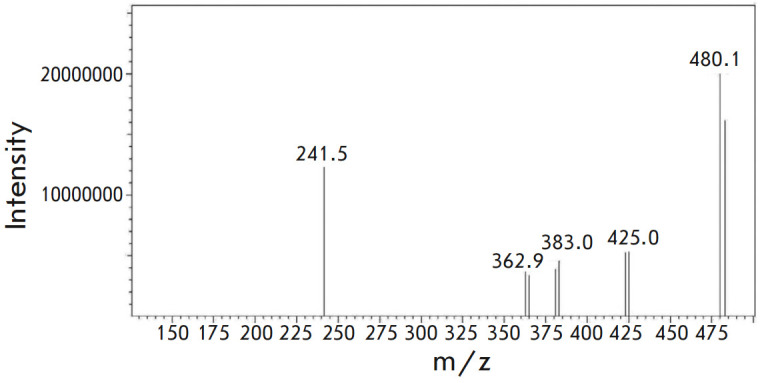
Mass spectrum of compound **1**

HRMS (ESI): Found m/z 480.1860 [M+H]; Anal. Calcd. for C_23_H_35_BrN_3_O_3_^+^ 480.1862 (*Fig. 5*).

**Elemental analysis**

Found: C, 49.89; H, 6.76; N, 7.48; Anal. Calcd.: C, 49.92; H, 6.56; N, 7.59; C_23_H_36_BrCl_2_N_3_O_3_.

**The solubility **of compound **1 **was determined in accordance with the General Pharmacopoeia Monograph (GPM.1.2.1.0005.15) [9]; it was inferred that 6-bromo- 1-methyl-5-methoxy-2-(1-piperidinomethyl)-3-(2-diethylaminoethoxy) carbonylindole dihydrochloride **1 **is an easily soluble compound.

**Cells**

A continuous kidney cell line of African green monkey (*Chlorocebus aethiops*) Vero E6 and a 293T cell line (a subclone of the transformed HEK 293 human embryonic kidney cell line, which is easily transfectable and maintains high levels of viral protein expression), as well as the L-929 mouse fibroblast cell line, were used in the experiment. All the cell lines were provided by the All-Russian Cell Culture Collection of the N.F. Gamaleya National Research Center for Epidemiology and Microbiology of the Ministry of Health of the Russian Federation.

**Animals**

Male outbred white mice (weight, 12.0–14.0 g) were procured from the animal husbandry of NEO Market OJSC (Veterinary Certificate No. 250 N0679392). The experiments were conducted in compliance with the rules outlined in the European Convention for the Protection of Vertebrate Animals used for Experimental and other Scientific Purposes [10].

The animals were allocated into groups (intact and four study groups, with three mice per group) by random sampling with allowance for body weight. Housing, feeding, and care for the animals, as well as termination of the experiments involving them, were performed in compliance with the rules of Laboratory Practice accepted in the Russian Federation [11]. Study Protocol No. 43 dated May 3, 2023 was reviewed and approved by the Ethics Committee of the study site.

**Viruses**

The pandemic strain of human coronavirus SARSCoV- 2 with infective activity of 106 TCID50/mL for Vero E6 cells (clinical isolate: hCoV-19/Russia/ Moscow-PMVL-12/2020 (EPI_ISL_572398)) and the murine encephalomyocarditis virus (EMCV), Columbia SK-Col-SK strain with a titer of 107 TCID/mL, were used. The viruses were procured from the State Collection of Viruses of the D.I. Ivanovsky Institute of Virology, N.F. Gamaleya National Research Center for Epidemiology and Microbiology, Ministry of Health of the Russian Federation.

**Quantification of the cytotoxicity of compound (1)**

The Vero E6 cell culture in Gibco DMEM (Thermo FS) supplemented with 5 vol.% FCS, L-glutamine (2 mM) and a mixture of antibiotics (150 U/mL penicillin and 150 U/mL streptomycin) were inoculated into assay plates in the presence and in the absence of compound **1 **and incubated at 37 ± 0.5°C for 96 h in an atmosphere of 5% CO_2_. The monolayer confluence and cell viability were assessed daily. The culture medium was then removed from the plates, and 100 μL of the PC medium (DMEM medium supplemented with 2% Gibco FCS (Thermo FS)) and 20 μL of the CellTiter 96® Aqueous One Solution Cell Proliferation Assay (MTS) (Promega, G3582) were added to the monolayer cell culture in each well [12]. The plates were incubated at 37 ± 0.5°C for 3 h; the results were recorded using a BIO-RAD automated reader at 490 nm using a 630 nm reference filter. The concentration of the solution of compound **1 **reducing the optical density at λ = 490 nm by 50% compared to the control was regarded as the 50% cytotoxic dose (CC50).

**Conducting the antiviral activity determination experiment**

A 24-hr monolayer cell culture prewashed with the PC medium and treated with non-toxic concentrations of compound **1 **(i.e., concentrations lower than the CC50 value) was used. Vero E6 cells were infected with the SARS-CoV-2 virus 60 min after addition of compound **1**. The following controls were used: positive control – cell culture infected with SARS-CoV-2 at different dilutions (from 10-1 to 10-7) without compound 1; negative control 1 – non-infected cell culture without compound **1**; negative control 2 – non-infected cell culture with 100 μL of the solutions of compound** 1 **at different concentrations. Each concentration of compound **1 **was tested in four parallel runs. The assay plates were incubated at 37 ± 0.5°C for 96 h in an atmosphere of 5% CO_2_ until the CPE of the virus was completely manifested in the viral control within the expected range. The antiviral activity of compound **1 **was determined visually under a microscope 96 h post-infection by inhibition of the CPE of the virus in the Vero E6 cell culture. The inverse of the final dilution at which CPE had developed was regarded as the viral titer. TCID50 was calculated using the Reed–Muench method for each concentration of the analyzed compound and control virus titer. The result was evaluated according to Δlgmax (the maximum decrease in the infective dose of the virus in the experiment compared to the control, expressed as decimal logarithms). The concentration of compound** 1 **reducing the virus titer by at least 1.5 lg was considered the minimum inhibitory concentration. The experiments were performed in three replicates to ensure statistically significant results.

**Quantification of the efficiency of inhibition of syncytium formation**

293T cells were co-transfected with a plasmid containing full-length S glycoprotein (pVAX-1-S-glycoprotein; Evrogen, Russia) and a GFP-encoding plasmid (pUCHR-IRES-GFP) using the Transporter™ 5 transfection reagent during 48 h. Next, compound **1** was added to the Vero E6 cell monolayer grown in 96-well plates at different concentrations and a suspension of 293T/S/GFP effector cells (3 : 1 cell ratio) was added to the wells. After 2 h, the number of syncytia formed was evaluated by fluorescence microscopy. The efficiency of inhibition of the cell–cell fusion induced by S glycoprotein SARS-CoV-2 compared to that in the control (without compound **1**) was assessed using the GraphPadPrism 5.0 software and presented as a percentage.

**Quantification of IFN-inducing activity**

Blood samples were collected from the decapitated animals into tubes without anticoagulants 2, 24, 48, and 72 h after a single intraperitoneal injection of compound **1 **at a dose of 121.2 μmol/mouse (70 μg/mouse) or 0.2 mL of distilled water (placebo, control without compound **1**). The IFN activity in mouse serum was quantified for the L-929 mouse fibroblast cell line. A three-day-old monolayer of a passaged L-929 cell line grown on medium 199 and DMEM (1 : 1) supplemented with 7% FCS, L-glutamine and antibiotics (150 U/mL penicillin and 150 U/mL streptomycin) was used. The serum IFN level was determined by titrating samples in the L-929 mouse fibroblast culture using the mouse EMCV as a viral indicator: the final IFN dilution protecting 50% of the cells against the cytopathic effect of 100 TCID50 of the virus was determined.

**Statistical analysis**

The half-maximal cytotoxic (CC50) and inhibitory concentrations (IC_50_) were calculated with the methods generally used in biological studies using the Microsoft Excel 5.0 and GraphPad Prism 6.01 software packages. The four-parameter logistic regression equation (menu items “Nonlinear regression” – “Sigmoidal dose-response (variable slope)”) was used as a working model for analyzing CC_50_. The four-parameter logistic regression equation (menu items “Nonlinear regression” – “log (inhibitor) vs. response (variable slope)”) was used for analyzing IC_50_. The selectivity index (SI) was calculated based on the data obtained according to the equation SI = CC_50_/IC_50_.

## RESULTS

**Quantification of the cytotoxicity of compound 1**

**Fig. 6:**
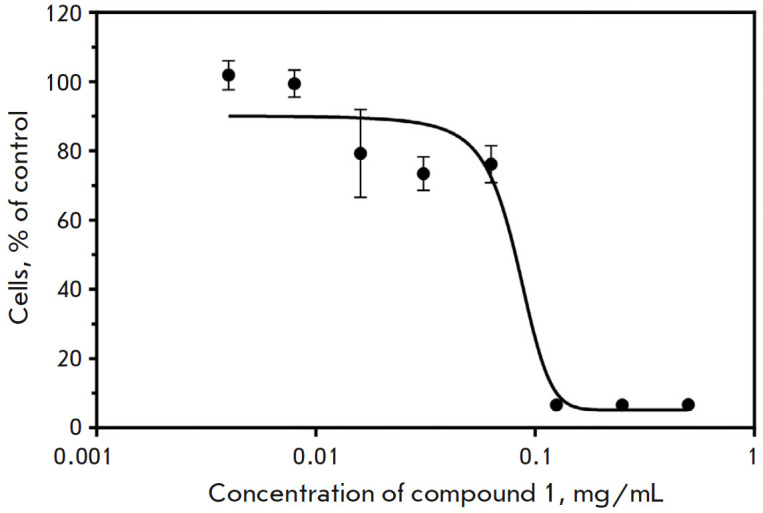
Determining the cytotoxic effect of compound** 1 **96 h after its addition to the Vero E6 cell culture (using MTS vital dye). CC_50_ = 83.32 μg/mL

The data obtained by studying the cytotoxic effect of compound **1 **on the Vero E6 cell culture using a MTS vital dye were used to plot an analytical curve. Using this curve, we determined the SS50 value for compound **1**. The concentration reducing the optical density value by 50% compared to the control was 83.32 μg/mL (144.30 μM) (*Fig. 6*).

**Quantification of the antiviral activity of compound 1**

The antiviral activity of compound **1 **was determined according to the decline in the infectious virus titer (TCID50/mL) in the Vero E6 cell culture (*Table 1*).

**Table 1:** The effect of compound 1 on the replication of SARS-CoV-2

Concentration of compound 1, µM (µg/mL)	TCID_50_	Virus control	Δlg_max_ the maximum reduction of the infective dose of the virus in the experiment compared to control (expressed as the decimal logarithm)
52.0 (30.0)	10^0^	10^6^	6
26.0 (15.0)	10^1^	10^6^	5
13.0 (7.5)	10^3^	10^6^	3

Note: TCID_50_ – 50% tissue culture infectious dose.

*Table * demonstrates that the analyzed compound exhibits a reliable dose-dependent antiviral activity* in vitro*, which is indicative of its specific action, and completely suppresses the replication of the SARSCoV- 2 virus at a concentration of 52.0 μM (30 μg/mL) (i.e. by 6 lgTCID50). In virological studies, the antiviral effect of drugs is usually considered satisfactory if Δlg TCID50 is ≥ 2.0 [13].

The IC_50_ value for the analyzed compound calculated using the GraphPadPrism 6.01 software was 1.84 μM (1.06 μg/mL). The selectivity index (SI) calculated as the ratio of CC50 to IC_50_ (SI = CC50/IC_50_) was 78.6.

**Studies focusing on the efficiency of inhibition of the syncytium formation induced by the SARS-CoV-2 spike protein (S glycoprotein)**

Additional studies were performed for syncytium formation mediated by the SARS-CoV-2 spike protein (S glycoprotein). Syncytium formation induced by the SARS-CoV-2 spike protein (S glycoprotein) was found to be inhibited by 89%.

**Quantification of interferon (IFN)- inducing activity of compound 1**

**Table 2:** IFN activity in mouse serum

Time after injecting compound 1 or placebo to mice, h	IFN titer (U/mL)	
	Compound 1 (70 µg/mouse)	Placebo (control without compound 1)
2	40	< -4
24	20	
48	20	
72	20	

The titration data are listed in *Table 2*.

Compound **1, **administered intraperitoneally at a single dose of 121.2 µmol/mouse (70 µg/mouse), was shown to exhibit an IFN-inducing activity.

## DISCUSSION

Discovered and described in the 1960s, the coronavirus failed to draw much attention, because it caused acute respiratory infections with a mild course [14, 15]. However, the COVID-19 coronavirus pandemic has changed our attitude towards coronaviruses and initiated a search for antiviral agents that would be effective against SARS-CoV-2. The search and development of drugs for controlling the pandemic coronavirus infection are tightly related to the point of application to viral replication and its effects during the treatment of patients. According to recent publications, the course of SARS-CoV-2 infection is divided into four stages associated with different requirements to drugs [16, 17, 18]. Thus, the pre-exposure stage is preferred for prophylactic immunization, use of neutralizing antibodies, and prophylactic antiviral drugs. Antibodies and intravenous and/or oral antiviral medications are effective during the next stage, after an individual has already been infected and viral replication is taking place. It has been established that a therapeutic effect is observed if anti- SARS-CoV-2 antibodies are administered within 10 days after the onset of symptoms [19, 20]; oral antiviral drugs can have an effect within 3–5 days after the onset of symptoms [17, 21]. The replication rate of the SARS-CoV-2 virus is known to increase approximately 3–5 days after the onset of clinical symptoms and then decrease within two or three days. The subsequent clinical events are associated with disruption of the immune response to SARS-CoV-2 [22]. However, in some patients, a recurrence of COVID-19 symptoms and presence of the virus were detected much later, indicating that the virus persisted and successfully continued to replicate in individual compartments during the later phase [23]. Therefore, it became clear that an oral antiviral and immunomodulatory small-molecule drug, which can be used during infection reactivation, is also needed to suppress viral reactivation during the late phases of the disease.

An extensive search across preclinical and clinical studies has identified a large number of compounds that exhibit anti-SARS-CoV-2 activity (low-molecular- weight compounds, monoclonal antibodies, peptide inhibitors, macromolecular inhibitors, as well as RNAand cell-based therapeutics) [24, 25, 26].

Unlike vaccines, the available antiviral chemotherapeutic agents inhibiting viral replication (including unusual nucleosides, and inhibitors of virus-specific proteins and enzymes) are generally effective against a broader range of pathogenic viruses. However, adverse effects are likely to develop during their use and resistant virus strains emerge thus leading to disease recurrence and exacerbation [27, 28].

Umifenovir (arbidol, the international name Umifenovirum) [29] is one of the commonly used antiviral drugs in Russia. It is included in the COVID-19 prevention and treatment guidelines [18]. According to Leneva et al. [30], umifenovir exhibits an antiviral activity and inhibits fusion of the viral envelope with cell membranes. Therefore, the virus cannot penetrate into the cell and its replication is suppressed. Umifenovir was shown to inhibit SARS-CoV-2 replication in Vero E6 cells [31, 32]. However, because of its low bioavailability and water insolubility, the therapeutic efficacy of arbidol is limited; so, it failed to assume a leading position among anti-COVID medications [33, 34].

The proposed drug candidate based on 6-bromo- 1-methyl-5-methoxy-2-(1-piperidinomethyl)-3-(2-diethylaminoethoxy) carbonylindole dihydrochloride will most probably preferentially be aimed at preventing the infection and inhibiting SARS-CoV-2 replication. At this very stage, viral replication and reproduction in the adjacent cells needs to be suppressed and the innate immunity has to be stimulated by receptor activation and induction of the interferon system. The ability of compound **1**, used at a concentration of 52.0 μM, to completely stop the replication of the SARS-CoV-2 virus in cells and the discovered effective mechanism of inhibition of syncytium formation induced by the spike protein (S glycoprotein) of the SARS-CoV-2 virus may apparently contribute to it. The revealed interferon-inducing ability of compound** 1 **may be indicative of its potential to activate the interferon system and the innate immunity, which also allows one to use the investigational drug to suppress the consecutive stages of immune dysfunction occurring in patients with COVID-19.

## CONCLUSIONS

Our findings demonstrate that the synthesized compound **1 **exhibits an antiviral effect against SARS-CoV-2 in *in vitro *studies. At a concentration of 52.0 μM, this compound completely inhibited the replication of the SARS-CoV-2 virus, with an infectious activity of 106 TCID50/mL. The concentration curves indicate the specificity of the action of the analyzed compound and show that the developed compound is rather promising and can be further studied *in vitro *in experimental animals. Due to its synthetic accessibility, high activity (IC_50_ = 1.06 µg/mL), and high selectivity index (SI = 78.6), compound **1 **meets the requirements for developing antiviral drugs for COVID-19 prevention and treatment.

## Abbreviations

WHO: World Health Organization
EMCV: encephalomyocarditis virus
IFN: interferon
IR: infrared spectrum
MS: mass spectrum
TCID50: 50% tissue culture infectious dose
CPE: cytopathic effect
Tm: melting point
FCS: fetal calf serum
1H NMR: proton nuclear magnetic resonance
CC50: 50% cytotoxic concentration of the compound
COVID-19: coronavirus disease 2019
DMEM: Dulbecco’s Modified Eagle Medium
GFP: green fluorescent protein
IC50: half-maximal inhibitory concentration
S glycoprotein: spike protein of the SARS-CoV-2 virus
SARS-CoV-2: severe acute respiratory syndrome caused by CoV-2 - Coronaviridae: Coronavirinae: Betacoronavirus: Sarbecovirus
SI: selectivity index calculated as a ratio of CC50 to IC50 (SI = CC50/IC50)
